# A novel toolbox to investigate tissue spatial organization applied to the study of the islets of Langerhans

**DOI:** 10.1038/srep44261

**Published:** 2017-03-17

**Authors:** Hoa Tran Thi Nhu, Rafael Arrojo E. Drigo, Per-Olof Berggren, Thomas Boudier

**Affiliations:** 1 Sorbonne Universités, UPMC Univ Paris 06, UJF, CNRS, IMT, NUS, Image and Pervasive Access Lab (IPAL), 138632, Singapore; 2 Bioinformatics Institute, Agency for Science, Technology and Research (A*STAR), 138671, Singapore; 3 Lee Kong Chian School of Medicine, Nanyang Technological University, 50 Nanyang Drive, Research Techno Plaza, Level 4, 637 553 Singapore; 4 Nanyang Institute of Structural Biology, Nanyang Technological University, Proteos, 61 Biopolis Drive, 138673, Singapore; 5 Rolf Luft Research Center for Diabetes and Endocrinology, Karolinska Institutet, Stockholm, Sweden

## Abstract

Thanks to the development of new 3D Imaging techniques, volumetric data of thick samples, especially tissues, are commonly available. Several algorithms were proposed to analyze cells or nuclei in tissues, however these tools are limited to two dimensions. Within any given tissue, cells are not likely to be organized randomly and as such have specific patterns of cell-cell interaction forming complex communication networks. In this paper, we propose a new set of tools as an approach to segment and analyze tissues in 3D with single cell resolution. This new tool box can identify and compute the geographical location of single cells and analyze the potential physical interactions between different cell types and in 3D. As a proof-of-principle, we applied our methodology to investigation of the cyto-architecture of the islets of Langerhans in mice and monkeys. The results obtained here are a significant improvement in current methodologies and provides new insight into the organization of alpha cells and their cellular interactions within the islet’s cellular framework.

With the development of new imaging techniques, such as single- and two-photon scanning laser microscopy and single plane illumination microscopy, the acquisition of volumetric image data from thick tissue samples is more common^1^. Though a lot of effort has been done on the automated analysis of cells or nuclei in microscopic images, the tools to analyze the spatial organization of tissues are limited. Analyzing the 3D organization of cells in tissue datasets is not common, and the measurements are mostly done on individual cells^2^,^3^,^4^ or with the tissue as a whole^5^.

Tissue analysis requires the identification of different cellular components and the computation of the physical interactions between them. In most cases the components are the cells themselves. Towards this goal, scientists first need to identify the location and identity of cells that make up a given tissue. Since clear cytoplasmic or membrane labelling is usually difficult to obtain in thick tissue samples, most studies rely on a nuclear labeling (e.g. DAPI) as a cellular identification approach. However, nuclei segmentation, especially in large 3D image datasets, is not trivial and remains an active research area among bioimage informaticians^6^,^7^,^8^. Furthermore, whole tissue analysis poses an additional challenge when segmenting cells within a crowded cellular environment. In this case, commonly used techniques for segmenting nuclei or cells are based on a “region-growing approach”^9^,^10^,^11^,^12^,^13^,^14^ and where FARSIGHT is the best example^9^. However, more complex procedures are based on different methods such as local curvature analysis^15^, region-growing and iterative thresholding^16^,^17^, level sets^18^ or a competition between different methods^19^. Finally, once the primary segmentation step is complete, scientists need to determine the identity of the segmented cells. Depending on the markers available, this step relies on (i) manual annotation of images, (ii) simple thresholding of nuclear or cytoplasmic content or (iii) a more complex supervised machine learning approach^16^,^20^.

An interesting tissue organization can be found in Islets of Langerhans. The islets of Langerhans form the endocrine part of the pancreas and are directly involved in the pathogenesis of diabetes^21^,^22^. The islet is a multi-cellular structure that houses insulin-secreting beta-cells, glucagon-secreting alpha-cells and somatostatin-secreting delta-cells among other rare cell types^23^. The islet’s main function is to maintain proper blood glucose levels at all times, which in turn is achieved by a coordinated action of the three-major cell-types in response to changes in circulating glucose levels^24^. Furthermore, an intricate network of vessels, nerves, autocrine and paracrine signaling loops supports proper islet development, survival and function and thus grants the islet the status of a complete “mini-organ”^24^. The cyto-architecture of rodent and primate islets is markedly different. The rodent islet is characterized by a relative majority of insulin-secreting beta-cells located at the islet core and surrounded by a mantle of glucagon-secreting alpha-cells and somatostatin-secreting delta-cells^23^. On the contrary, the primate islet (i.e. monkey and human) displays a heterogeneous distribution of all cells^23^,^25^. Therefore, to fully understand human islet physiology and pathophysiology there is a need to depart from mouse-based models and move towards a closer surrogate of human islet physiology, namely the monkey islet.

Previous works have tackled the problem of analyzing the islet cyto-architecture using a large bank of islet sections. Striegel *et al*.^26^ analyzed the clustering of beta-cells in human islets while Hoang *et al*.^27^ and Kilimnik *et al*.^28^ analyzed the spatial organization of alpha and beta-cells using manual detection of different islet-cell types and computed the cellular direct interactions based on a complex computation of distances and angles. More recently Poudel *et al*. developed a tool to analyze different cell-type composition of islets but only in 2D and they did not investigate the cellular network^29^. No attempt has been made to identify the interaction network of different islet cells in 3D. Understanding the islet three-dimensional organization and the configuration of supporting regulatory signaling networks is an important step towards understanding the setup of paracrine signaling pathways that support islet function^21^.

Here we bring forth a novel toolbox containing all the necessary methods for nuclei segmentation, cell identification and analysis of cell interaction in 3D. More importantly, this toolbox is integrated into the widely-used ImageJ platform as an easy-to-use plug-in. We applied this toolbox to investigate current and novel aspects of the unique architecture of the pancreatic islet of Langerhans.

## Results

**Figure 1:**
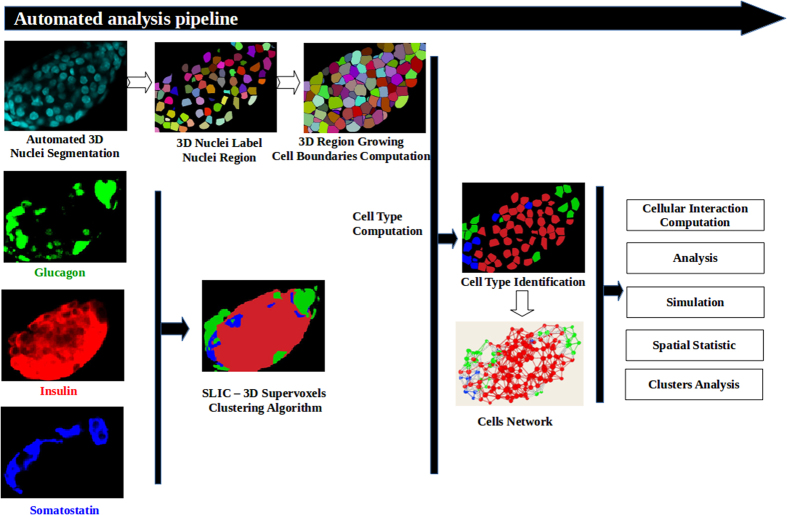
Workflow of the analysis. Nuclei are segmented and cell boundaries computed from the DNA-label channel. The images corresponding to the different labels of the different cell types are combined together in a SLIC image (see text for details). This combined image is used to compute each cell type. Finally, the cell interaction network is created using the cell boundary and cell type information.

All segmentation and analysis algorithms were implemented in Java as ImageJ plugins^30^ and all filters and analysis tools are based on our homemade 3D library for image analysis^31^. The tools presented here cover different aspects of spatial analysis of tissues in 3D, ranging from nuclei segmentation to analysis of different aspects of cellular interaction and network mapping. We applied our tools to the study the complex multi-cellular structure of mouse and monkey islets of Langerhans (Fig. 1). The files for the toolbox can be found at: http://imagejdocu.tudor.lu/doku.php?id=plugin:3d:tissue_organization_toolbox:start.

### Datasets

We applied our methodology to analyze 3D confocal image stacks of islets of Langerhans in whole or serial histological pancreas sections from two different species: C57/BL6 mouse (8 weeks of age, n = 6 animals and 8 stacks) and monkey (Macaca fascicularis, 9–12 years of age, n = 6 animals and 6 stacks). Detailed information for the islets used as testbeds for validation of our toolbox can be found in Table 1. Additional information about the sample preparation and imaging protocols is shown in the methods section. Besides thick tissue sections, we also analyzed 2 whole mouse islets (Mouse 4 and Mouse 6) that were obtained from optically cleared tissues using SeeDB and imaged with 2-photon microscopy^32^.

### Nuclei segmentation

In homogeneous tissues, cells and nuclei tend to have roughly the same shape and size, while also appearing in a tightly packed organization with proximity between nuclei. In this work, we used DAPI staining to label the cell nucleus. First, the nuclear label signal was filtered to reduce noise and enhance structures based on their size. We then used our spot segmentation procedure to segment the nuclei (Fig. 1 and methods), where the local maxima of the filtered image are detected and used as seeds for local segmentation^33^.

**Figure 2:**
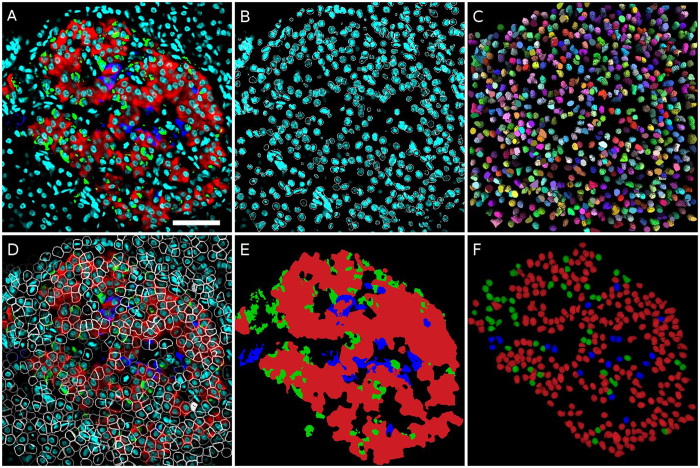
Results of nuclei segmentation and cell type computation for the monkey1 dataset. (**A**) Merged image of raw data, only one slice is displayed (red = insulin, green = glucagon, blue = somatostatin, cyan = DAPI). (**B**) DAPI channel with overlaid contours of detected nuclei. (**C**) 3D visualization of segmented nuclei. (**D**) Merged raw image with overlaid contours of computed cell boundaries. (**E**) 3D SLIC supervoxels clustering result (red = insulin, green = glucagon, blue = somatostatin) (**F**) 3D visualization of computed cell types (red = beta-cells, green = alpha-cells, blue = delta-cells). Scale bar, 50 *μ*m.

This nuclei segmentation approach allowed us to identify and segment correctly approximately more than 97% of total nuclei in all datasets (Fig. 2A–C and Table 2). In addition, we observed a low false-positive rate of 106 objects out of 8837 nuclei and a low false negative rate of 257 objects (Table 2) for our validated dataset (Table 2). We obtained a final F-measure of approximately 97.7%, which is comparable or better than other algorithms (Table 2, Supplementary Fig. 3). Most of the false-positive detections are due to the occasional observation of nuclei that are split into two different objects. We also obtained a low false negative detection rate for nuclei, and that corresponded to nuclei that were near each other and below the resolution potential of the confocal microscope in the X-Y and Z-axis (Tables 1 and 2).

### Cell phenotype computation

Due to the limited number of possible markers that could be used in a single immunofluorescence experiment, we did not stain the plasma membrane in our study. Instead, we used the nuclear volume as the theoretical core of the cell and then predicted the maximum likelihood of the cell “zone” by extending the nuclei boundaries according to an average cell size (4 *μ*m, Fig. 2D). To avoid the potential overlap of two close nuclei, we added a watershed procedure to ensure the exact separation between two nuclear volumes (Fig. 2B,D).

For our application, the islets of Langerhans is composed of three main types of endocrine cells, namely alpha-, beta- and delta-cells. Therefore, our mouse and monkey datasets were composed of three different channels that represent glucagon, insulin and somatostatin markers (Figs 1 and 2A). All three markers are cytoplasmic and are sufficient to determine the cell identity. The labelling is quite dense throughout the cytoplasm and no clear separation between the different cells was visible (Fig. 2A).

To reduce the complexity of the number of voxels in each dataset, we clustered the voxels from the different images containing the specific cell markers (i.e. insulin, glucagon and somatostatin) into regions and combined this information into one single image. This was achieved using a supervoxels clustering approach^34^ resulting in a single 3D image where islet-cells are represented by regions containing voxels of similar values for all three channels (Figs 1 and 2E). Next, each region was assigned a cell phenotype based on the most abundant label with the highest average median intensity value. If the intracellular signal intensity for all three channels was below a predefined threshold, the region was classified as “unlabeled” (Figs 1 and 2F). In the case of small regions that are labelled (but not belonging to the “unlabeled” type) inside a cell zone, and below a predefined minimum threshold, the cell was also assigned to the “unlabeled” category (Table 3). The application of this algorithm on mouse and monkey islet image datasets correctly identified cells with an accuracy of more than 97% (Table 3).

Next we applied our toolbox to mouse and monkey islets and investigated their endocrine cell composition. Here we observed a mean percentage of alpha-cells (*P*_*α*_) is 18.2% in mouse and 21.2% in monkey islets; for beta-cells, the percentage (*P*_*β*_) is 76.4% in mouse and 66.9% in monkey islets, and for delta-cells (*P*_*δ*_) is 5.4% in mouse and 11.9% in monkey islets (Table 4). The average cellular composition and cell types frequency of mouse and monkey islets used in this study are within the described values in the literature^23^.

### Analysis of direct cell-to-cell interactions

Once all islet-cells were properly segmented and identified, we built a network of interacting cells using their cell zones. If two cell zones are touching we build an link between the two cells and define them as interacting. First, when analyzing intact mouse islets (i.e. mouse4, and mouse6 in Tables 1, 2, 3, 4 and Supplementary Table 1–5), we observed a higher number of direct interactions per islet-cell (mean of 7.48, 8.98, 9.94 interacting cells of alpha, beta and delta cells respectively - Supplementary Table 1) what suggests that analyzing thin and individual histological sections may underestimate the number of connections in a tissue dataset. Second, we computed the relative proportion of different cell types interactions, defining homotypic interactions between similar cell types (alpha-alpha (*P*_*αα*_), beta-beta (*P*_*bb*_) and delta-delta (*P*_*δδ*_)), and heterotypic interactions between two different cell types (alpha-beta (*P*_*αβ*_), …). We found more homotypic beta-beta interactions in mouse datasets than in monkey datasets, and approximately same proportion of alpha-alpha interactions in mouse and monkey datasets (Supplementary Table 2).

**Figure 3:**
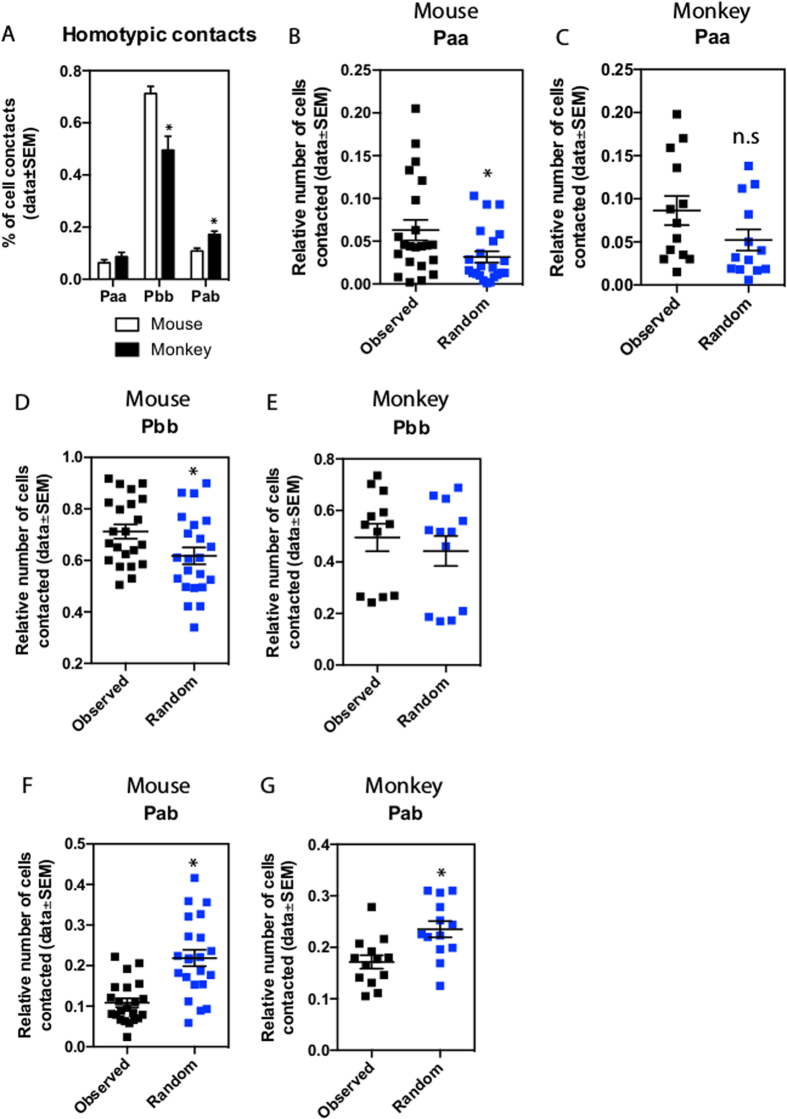
Relative proportions of direct cellular interactions between the two main cellular types alpha- and beta-cells for the extended datasets (6 mice, n = 22 datasets; 6 monkeys, n = 12 datasets). (**A**) Homotypic contacts in mouse and monkeys datasets (*) denotes significant difference. (**B–G**) comparison of cellular interactions between extended mouse and monkey datasets and random models (*) denotes significant difference.

In order to investigate further the different organisation of islets of Langerhans in mice and monkeys we restrained our analysis on the two major cell types alpha and beta, and added more datasets for this analysis (Fig. 3). Due to the heterogeneous organization of primate islets with a lower relative number of beta cells, we observed a significantly lower *P*_*ββ*_ of 49.4% (p < 0.05 vs mouse, Fig. 3A). *P*_*αα*_ was not significantly different from mice at 8.6% (Fig. 3A). Next, we investigated the number of direct contacts between alpha- and beta-cells in mouse and monkey islets. Here we observed that monkey islets have a significantly higher percentage of *P*_*αβ*_ than mouse islets (Fig. 3B, 17.1% vs. 10.8%, p < 0.05).

Following the work from Kilimnik *et al*.^27^,^28^, we computed the percentage of direct cellular interactions that should occur in a random organization. If alpha-cells are organized randomly, the theoretical probability of observing an interaction between two alpha-cells is determined by 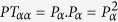, where *P*_*α*_ represents the probability of a cell to be an alpha-cell and 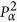 the probability that the first and also the second randomly chosen cell are alpha-cells. The same logic applies to the case of beta-cells or between alpha- and beta-cells. In the latter case, when two random cells are chosen the probability that the first cell is an alpha-cell is determined by *P*_*α*_ and the probability that the second cell is a beta-cell is given by *P*_*β*_. Therefore, the theoretical probability of a random alpha- and a beta-cell to interact is determined by *P*_*α*_. *P*_*β*_. However, the possibility that the first cell is a beta-cell and the second cell is an alpha-cell must be considered as well, and thus the final equation is 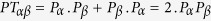. If the interaction between cells of the same type is not random, one should observe higher values than those generated randomly. In this same setting, the observed heterotypic interactions would be lower than random values. We designed a randomization procedure of the cells inside the tissue and compared the results of the proportion of interactions between our randomised models and the theoretical proportions as presented above (Supplementary Fig. 6). We did not observe differences in the results (data not shown), so we can conclude that our randomized model will effectively capture a random organisation of the tissue.

When compared to random models our results show that mouse islets have significantly higher homotypic contact frequencies than what should occur in a random organization (Fig. 3B and D, observed vs. random). We observed a similar, but not statistically significant trend in monkey islets (Fig. 3C,E). These results indicate that in the mouse islet alpha- and beta-cells are not randomly organized. Interestingly, in mouse and monkey islets, the observed number of contact frequency between alpha- and beta-cells was significantly lower than the values obtained with a simulated random distribution (Fig. 3F,G).

### Analysis of indirect cell-to-cell interaction

Next, we investigated the framework of the indirect cellular interactions in mouse and monkey islets. Briefly, this was calculated with the following logic: when a given islet-cell “*X*” contacts directly a predetermined “*reference*” cell, “*X*” will be at distance equal to 1 from the “*reference*” cell (Supplementary Fig. 1, white cell). If another islet-cell “*Y*” contacts islet cell “*X*”, “*Y*” will then be at a distance value of 2 from the “*reference*” cell (Supplementary Fig. 1, dark purple cells). This mechanism allows the computation of relative distance maps for any cell or group of cells within our dataset in 3D. Using alpha- and beta-cells as test platforms, we computed the cell distances between beta-cells and the closest alpha-cell. We observed that, in both mouse and monkey islets, most of the beta cells are less than 4 cells away from the closest alpha-cell (Supplementary Table 3). When comparing the distances from observed and randomised data we observed that in a random organisation of alpha-cells, beta-cells tend to be closer to alpha-cells. This indicates that alpha-cells tend to be clustered.

Following the application of the randomization procedure to alpha-cells, we performed a more complex analysis by comparing the number of indirect beta-alpha interactions and of cell distances in both observed and randomized patterns (Supplementary Table 3). For both mouse and monkey islets, we observed a higher number of beta-cells with large cell distances to alpha-cells in the observed datasets than in our randomized models for distance greater than or equal to 2 (Supplementary Table 3). This observation supports the concept that alpha-cells are organized in a cluster-like pattern. This is specially represented by the well-known mantle organization of alpha cells in mouse islets. However, this difference is less striking in monkey, suggesting that alpha-cells are more uniformly distributed throughout the islet and thus likely explain the higher frequency of alpha-beta contacts (Fig. 3 and Supplementary Table 3).

### Analysis of spatial organization

The focus of investigating the spatial organization of a tissue is to understand the distribution and patterning of its cellular framework. This goal can be achieved by testing whether the cellular organization is random and applying statistical computation to confirm it. To address this point, we used the classical spatial statistics F- and G-functions^35^. These functions are based on a histogram computation of distances between points, usually corresponding to the location of molecules^36^. The G-function is based on the distances between two closest points and reflects the existence of clusters. The F-function is based on the closest distances between reference and points of interest, thus reflecting the existence of “geographical voids”. Using this concept, the known distances for our observed data can be compared to distances from the same number of randomly generated points (in our case the islet alpha-cells). This comparison is performed inside the islet space where all the cells are found. The result is a normalized index between 0 and 1 termed Spatial Descriptor Index (SDI)^35^. This index expresses the rank of the observed data values within the simulated models. A rank between 0.05 and 0.95 indicates that the observed data is within the 95% confidence interval of all simulated random data. Therefore, in this scenario the observed values are not significantly different from a random organization pattern.

To investigate further the spatial organization of alpha-cells, we applied this concept on mouse and monkey islet datasets. We observed SDI values of F- and G-function in mouse (Supplementary Fig. 2A,B) and monkey islets (Supplementary Fig. 2E,F) outside the 0.05–0.95 interval (Supplementary Table 4), which indicates that alpha-cells are organized in a non-random distribution pattern. These results also support our previous observations (Supplementary Table 3) that alpha-cells are organized in clusters in both mouse and monkey islets. Of note, our simulations of a randomized organization generated expected results with SDI values within the 95% confidence interval (Supplementary Table 4 and Supplementary Fig. 2).

Given the cellular architecture of the islet and the importance of paracrine signaling factors secreted by alpha- cells^24^, we hypothesize that the interactions between neighboring cells is more significant than the shortest distance (i.e. Euclidean distance) between two islet cells. Therefore, we modified the classical F- and G- functions to use cell distance instead of the Euclidean distance parameters. Using this approach, we observed similar results and where most SDI values were outside of the 95% range (Supplementary Table 4). This observation indicates again a non-random organization of alpha-cells in both mouse and monkey islets. As expected, a randomized distribution of alpha-cells resulted in SDI values for F- and G- functions were within the 95% confidence interval (Supplementary Table 4).

Finally, we sought to characterize the composition of the alpha-cell clusters. For analytical purposes, a cluster was defined as a single cell or a set of cells directly connected by a cell distance equal to 1. In mouse islets, alpha-cell clusters ranged in size (1 to 93 cells, mean of 5.43 cells per cluster, (Supplementary Table 5)), while monkey islets had smaller alpha-cell clusters (1 to 27 cells, mean of 2.85 alpha-cells per cluster, (Supplementary Table 5). We also found most of the SDI values for alpha-clusters in the interval between 0.05–0.95 suggesting a non-random organisation at this cluster level, however this trend is less clear than at the cell level.

## Discussion

### Nuclei segmentation

Although nuclei segmentation has been studied for many years, no definite tools or algorithms are available, especially for thick tissue samples. One of the most efficient tools is FARSIGHT^9^, which did not present a better performance in comparison to our algorithm (Supplementary Fig. 3). The main originality of our approach is the use of a filtering to enhance structures based on their size with a high degree of specificity (Fig. 1 and Table 2). Moreover, this filtering allows us to discard any eventual intensity variation between nuclei and within the nuclei and contributes to a high detection efficiency observed in our dataset.

### Computation of cell zones

The computation of marked cell zones is an essential part in the analysis of the spatial organization of any given tissue. In our dataset, the membranes of islet cells were unlabeled and thus we used watershed approach to separate nuclei and compute plausible cell zones around each nucleus (Fig. 2D). Nevertheless, our observed results indicate a high accuracy in cell phenotype identification (Table 3). Subsequent analysis of cellular interactions confirmed previous results describing homo- and heterotypic cellular interactions in mouse and monkey islets (Fig. 3 and Supplementary Table 2). Moreover, 3D analysis of an intact islet made possible by tissue clearing yielded the largest number of possible cell-cell interactions (mouse4 and mouse6, Supplementary Table 1) when compared to single or serial sections datasets. This suggests that analysis of thin tissue sections is insufficient and does not provide a real account of a tissue spatial organization, thus strengthening the need for real 3D imaging and analysis. The results described here for islet-cell type distribution and cell-cell interaction are comparable to the literature^23^ and thus validate our computational approach.

In the present work, our model defines the interaction between two cells as direct physical contacts and do not consider the presence of important extracellular matrix proteins or blood vessels, both of which are important factors for islet-cell function. In future applications, we propose to include the identification of blood vessels and the analysis of the spatial relationship between islet-cells and the vasculature^37^,^38^. This is further underscored by recent studies indicating key anatomical and functional differences between mouse and human islets and their microvasculature^39^,^40^.

### Cell identity computation

The computation of a given cell type depends strongly on the quality of the imaging data and information available that allows the de-facto identification of a cell type. In our case, all markers used here are bona-fide markers of distinct endocrine cell types in the islet (Methods and Figs 1 and 2). Accurate cytoplasmic labeling is a challenging task to achieve mainly due to the thickness of the tissue and the variability of the staining methods itself. To compute the cell identity and the cell-cell interactions, we must first stipulate the cellular extension. In our case, since we were investigating 3 different cell types in addition to the nuclear stain (i.e. DAPI), it was quite challenging to add a fifth dye to stain the cellular membranes. Therefore, we assumed a central position of the nucleus within the cell and that the cytoplasmic volume expands only locally around the nucleus volume. Next, we used the classical watershed separation approach to compute a Voronoi diagram around the segmented nuclei. We however modified the watershed algorithm to constrain the Voronoi zones not to extend farther than a predefined radius from the nuclei boundaries. Finally, to process the three different labels altogether, we observed that the supervoxels clustering approach^34^ was the most robust and allowing a correct clustering for all the three channels. It is worth noting that while this approach was successful for our datasets, it is possible that for other applications investigating other cell types (e.g. neurons) a different combination of tools may be necessary^20^.

### Analysis of spatial organization

The main challenge in investigating the spatial organization is to identify robust and adapted descriptors. Classical descriptors such as SDI for F- and G- function (Supplementary Table 4 and Supplementary Fig. 2) are very well adapted for spots analysis, where objects distribute virtually anywhere in the enclosing structure. However, there is a difficulty in adapting this approach to whole tissue analysis where the location of objects is quite constrained and where structural voids (e.g. blood vessels) may exist inside the structure. We then proposed to use the same framework but we constrained the positioning of objects to cell locations. We also investigated cell distances instead of Euclidean distances (Supplementary Fig. 1 and Supplementary Table 3, 4), although we observed similar results using either distance parameter. However, for more complex cell networks with a non-convex topology, the cell distance analysis may become more effective in correctly assessing the organization of cells.

### The Cyto-architecture of Islets of Langerhans of mice and monkeys

By identifying and mapping the position of all three-major islet-cell types, our toolbox was validated by confirming known underlying differences in the cyto-architecture of mouse and monkey islets (Fig. 3 and Supplementary Table 2 (ref. 23)). We observed that while mouse islets are rich in homotypic contacts, monkey islets display a more heterogeneous islet-cell distribution that results in more heterotypic contacts between islet-cells (Fig. 3 and Supplementary Table 2). Interestingly, alpha-cells in both species tend to be organized in a non-random cluster organization and which leads to a non-random pattern of contacts between alpha- and beta cells (Fig. 3). This data suggests that alpha-cells are in specific areas within the islet, which may be set during the developmental period^41^ and underscore the potential paracrine role that alpha-cells have in islet physiology and diabetes^21^,^42^,^43^,^44^,^45^. Furthermore, we noted a subtle trend suggesting that alpha-cell clusters are not randomly organized in mice – representing the peripheral alpha-cell mantle - while the opposite is observed in monkeys.

In conclusion, in the present work we demonstrate the development of new digital tools to visualize, analyze and compare the different organizations of multicellular structures (e.g. tissues) and used the mouse and monkey islet as a proof of concept for such methodology. Our toolbox provides an easy-to-use software that can automate and standardize the data collection on islet morphology in an unbiased manner. The fundamental differences between rodent and primate islets described here support the hypothesis that the physiology and paracrine signaling architecture of rodent and primate islets are different and that alpha-cells are not randomly allocated within the islet ultra-structure^23^. This study highlights the differences between mouse and primate islets and points towards a shift towards islet studies focused on monkey and human islets for meaningful understanding of diabetes patho-physiology^22^,^46^,^47^,^48^.

## Materials and methods

All statistics were done using ANOVA method with the Graphpad Prism V6.0 software. Some figures were done using FigureJ^49^.

### Immunohistochemistry of mouse and monkey pancreatic islets of Langerhans

All animal procedures were approved by the Institutional Animal Care and Use Committee (IACUC, protocol number 2013/SHS/816) of the SingHealth system. Experiments were carried out in accordance to experimental guidelines established by the Singhealth Experimental Medicine Center (SEMC), which is an AAALAC accredited facility. Immunohistochemistry of mouse or monkey pancreas was performed as previously described^23^. Briefly, the pancreas from a 5-week-old wild type C57 mouse was dissected, rinsed in phosphate buffered solution (PBS) and fixed in 4 paraformaldehyde for 12 hours. Monkey pancreases were from animals used in other terminal studies. Following the fixation step, the pancreas was rinsed in PBS for 5 minutes and cryo-protected in a 30 sucrose solution for 12 hours. Next, using a razor blade, the pancreas was trimmed to smaller pieces (approx. 2 × 2 *mm*), embedded in freezing tissue medium FSC 22 (Leica Biosystems, Australia) and stored at −80 °C. Next, the pancreatic tissue was sectioned in 30 *μm* thick sections and placed on slides. Slides were air-dried for 1 hour on the bench top protected from light and rinsed 2 × 5 minutes in PBS. Next, slides were blocked and permeabilised with a blocking solution (10% fetal bovine serum and 0.3% triton X-100 in PBS) for 1 hour. Following the blocking step, samples were incubated overnight with the following primary antibodies: guinea pig anti-insulin (beta-cells, 1:200, Dako, California), rabbit anti-glucagon (alpha-cells, 1:200, Sigma Aldrich, USA) and rat anti-somatostatin (delta-cells, 1:200, Millipore, USA). After incubation, slides were rinsed 4 × 10 minutes in PBS. Next, slides were incubated for 1 hour with the following secondary antibodies: donkey anti-guinea pig AlexaFluor 568 (1:400, Invitrogen, USA), donkey anti-rabbit AlexaFluor 488 (1:400, Invitrogen, USA) and donkey anti-rat AlexaFluor 647 (1:400, Invitrogen, USA). Cell Nuclei was stained with DAPI (1:400, Invitrogen, USA). After incubation, samples were rinsed 4 × 10 minutes in PBS, dried and mounted for microscopy. Clear tissue samples were prepared according to Ke *et al*.^32^.

### Confocal microscopy and image acquisition

Single photon confocal microscopy images of islets of Langerhans were acquired using an upright Leica SP8 confocal microscope (Leica Microsystems, Germany) equipped with a white light laser and hybrid detectors (HyDs). The following excitation (Ex) and emission (Em) parameters were used for image acquisition: DAPI Ex-405nm and Em-432/481 nm; AlexaFluor 488, Ex-498nm and Em-507/559 nm; AlexaFluor 568 Ex-577nm and Em-588/681 nm; AlexaFluor 647 Ex-647nm and Em-656/762 nm.

### Image processing and analysis

All implementations were done in Java as ImageJ plugins using our home-made tools and library for 3D processing and analysis. Datasets and source code are available on demand. A wiki page, with detailed instructions on how to use the toolbox along with a sample dataset was created at: http://imagejdocu.tudor.lu/doku.php?id=plugin:3d:tissue_organization_toolbox:start.

#### Nuclei segmentation

Images corresponding to nuclear labeling (DAPI) were first processed by 3D median filter with a radius of 4–4–2 in *X*–*Y*–*Z* to reduce noise and homogenize intensities inside the nuclei. Images were then filtered by a 3D band pass filter^50^ with specific size interval of 20–28 pixels corresponding approximately to 6–7 *μm*. This is the approximate diameter of the average nucleus in our dataset. The resulting image is a 32-bit image; which is then scaled to a 16-bit image. Our plugin 3D Spot Segmentation^33^,^51^ was used to segment the nuclei with a local maxima in a radius of 4 voxels extracted from the band pass filtered image. A threshold is set to only detect local maxima corresponding to nuclei regions (threshold value was set to 30,000 for both mouse and monkey dataset images^33^). Next, we computed the 3D radial distribution of mean intensity values in growing concentric layers around each seed. From this radial distribution, we could fit a Gaussian curve and a cut-off is computed on the Gaussian curve to a specific standard deviation value (here set to 1.8). The computed value on the Gaussian fitting is then used as a local threshold for a region-growing algorithm around each seed. A 3D watershed is also performed to ensure the segmented objects will not propagate to neighboring seeds. A manual editing was then performed when necessary to correct few errors of segmentation, using the 3D Manager tool from the 3D ImageJ suite^31^,^51^, and to assess the accuracy of the segmentation. Split nuclei were merged, and merged objects were split where possible.

### 3D cell phenotype computation

The automated computation of the cells phenotypes comprises two steps: (i) the three markers are combined into regions using a supervoxels clustering approach, and (ii) cell zones are computed around each nucleus based on a watershed approach. Finally, the phenotype is determined by computing the most abundant marker inside the cell zone. In order to reduce complexity of the three different labels, we merged the information from our three cytoplasmic markers (namely insulin, glucagon and somatostatin) into one single image using a clustering approach. After a 3D median filtering with radius of 4–4–2 in *X*–*Y*–*Z*, we normalize the images by setting the values interval in each channel from 0 to 255. We then cluster the voxels in the three channels using the SLIC (Simple Linear Iterative Clustering) approach^34^. Basically, the image is first divided into square zones of similar size *R*, where the size is chosen between 100 and 300 pixels according to the size of the nuclei/cells. The median values of each channel are computed inside each zone. For each zone, we iterate over all the voxels *V* within a radius of 2 * *R* around the centre of the zone. We compute the distances, both in Euclidean and intensity space, *d*_1_ and *d*_2_ between the voxel and the centre of the zone as follows:


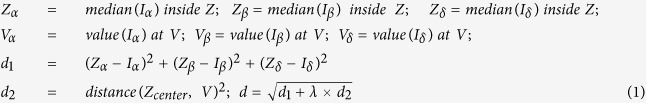


where *I*_*α*_, *I*_*β*_ and *I*_*δ*_ are the three images corresponding to the three labellings, *Z* is a zone, *V* is a voxel, *d*_1_ is the distance in the signals space, and *d*_2_ is the distance in the Euclidean space. The *λ* parameter controls the stiffness of the zone, in our study we chose a value of 10000. The algorithm is then quite straightforward and before each round of iteration the distance *d* is set to an arbitrary maximum value for all voxels. For each zone, we iterate over neighboring voxels in radius 2 * *R*, where if the distance *d* is lower than the previous distance set for the same voxel, we assign this voxel to this zone and update its current distance *d*. Each zone is then updated according to its assigned voxels and this process is iterated 10 times. Post-processing is performed to detect change in connectivity of the zones and to merge eventual disconnected zones to the closest one. We finally assign the type of each zone to the channel having the highest median value inside the zone. If all the median values inside the zone are lower than predefined thresholds, the zone is assigned to the unlabeled type. An image is then created with the voxels having the value of their corresponding zone type.

#### Cell zone computation - watershed separation

Based on the segmented nuclei images, we can define zones around each nuclei using a watershed separation. First, a 3D distance map is computed on the background of the segmented image of all nuclei. The resulting image is a 3D image where the value of each voxel represents the minimal calibrated distance to the closest nucleus boundary. Note that our 3D distance map implementation works using calibrated unit and hence take the anisotropy in Z into account. The distance map image is then inverted so highest value are close to nuclei boundaries. Then a typical watershed procedure is applied. Basically, all pixels are put into an ordered list and processed sequentially. First pixel, with the highest value, is processed, and the software performs a search on the pixel’s 3D neighborhood for voxels already assigned to a nucleus. Next, non-labelled voxels from the pixel’s neighborhood are added to the list based on their distance map value. In case two labels are encountered in a neighborhood, the voxel that is at a frontier between two zones is assigned a specific label. Then a mask is applied and set to 0 for voxels whose value in the distance map image is greater than the defined radius.

To detect the cytoplasmic signal with high specificity we look for markers in the SLIC image around the nuclei boundary. Here we define two distances: (1) the inner distance, from the nucleus boundary towards the inside of the nucleus and that detects fluorescence signal that may appear inside the segmented nucleus; (2) an outer distance from the nucleus boundary towards the outside of the nucleus. The volume of the compartment delimited by these two radii is constrained to stay inside the corresponding cell zone. In our experiment, we used the values 3 and 2 for the inner and outer radii. We compute the number of voxels for all 3 markers inside the compartment using the previously computed SLIC image. We then assign the cell-type (i.e. beta, alpha or delta) based on the signal intensity of the most abundant label. However, if the signal intensity inside the constraint compartment is below a threshold, the cell is assigned as *unlabeled*.

### Cells network

We modeled the network of cellular interactions as a graph where we used the cellular information (nuclear and cell-zones volumes) as the graph vertices. Edges are created between interacting cells. The interaction between two cells is defined as two cells having voxels of one cell touching a voxel of the other cell. The parameters for this computation considers a 26-voxel neighboring definition. In each image the different computed cell-zones have a specific label. For each cell-zone we iterate over the boundary voxels and compute the labels found in a 3 × 3 × 3 voxel neighborhood. If a voxel with a different label is found, an edge is added to the graph between the reference cell and the cell owing the neighboring label. For the analysis of cell distances, the distance values are computed within the graph using the Dijkstra's shortest path algorithm^52^. Using our tools visual display of 3D network is done within ImageJ using the ImageJ 3D viewer plugin^53^.

### Randomizing procedure

The randomization procedure used simply shuffles the cell types and do not actually moves the cells from their geographical position. In our case study, we selected the first alpha cell and then randomly chose another cell in the islet structure (e.g. another alpha, or a beta or a delta-cell). In the case the selected cell is not an alpha-cell we simply swap the two cell types. We repeat this procedure for all alpha-cells. Swapping the type of two cells will automatically change the number of direct interactions for the two cell types without affecting the number of direct interactions for the third cell type. These changes are in accordance with the theoretical values for direct interactions upon a random model.

### Analysis of spatial organization and cluster analysis

For the analysis of spatial organization, we followed the methodology of Andrey *et al*.^35^,^36^ and used the 2D/3D spatial statistics ImageJ plugin as a framework. However, we made a specific version for tissue analysis by modifying it to simulate random positions only at the position of existing cells (either alpha, beta or delta cells). For the G-function we compute the distances between an alpha-cell and its closest one. This is done first in the observed data and secondly in the randomly distributed alpha-cell organization. We compute the cumulated distribution function (cdf) for both observed and randomized data. From the randomized data, we compute first the average cdf from 100 randomized organizations followed by a computation of the confidence interval on a different set of 100 randomized organizations. The Spatial distribution index (SDI) is computed as the maximum difference between the cdf of the observed data and the average cdf of the randomized data. The same procedure is applied for the F-function, except that the computed distances are different. For the F-function, the computed distances are between reference points and the closest point. In our case, the positions of cells are chosen as reference points. We then modified the procedure further to allow the user to use cell distances and not only Euclidean distances. In our study, we use a set of 100 random distributions to compute the SDI value. For the cluster analysis procedure, for each cluster, we simply remove from the graph the cells comprising the cluster and replace them by one node having as coordinate the center of the cluster. We then run the spatial analysis procedure on the modified network.

## Additional Information

**How to cite this article**: Tran Thi Nhu, H. *et al*. A novel toolbox to investigate tissue spatial organization applied to the study of the islets of Langerhans. *Sci. Rep.* **7**, 44261; doi: 10.1038/srep44261 (2017).

**Publisher's note:** Springer Nature remains neutral with regard to jurisdictional claims in published maps and institutional affiliations.

## Supplementary Material

Supplementary Information

## Figures and Tables

**Table 1:** Origin and characteristics of mouse and monkey pancreas datasets used in this study

Species	Name	Size (XYZ)	Calibration (XY-Z) in nm
Mouse	Mouse1-a*	1024 × 1024 × 36	378.4 × 800.1
	Mouse1-b*	1024 × 1024 × 48	378.4 × 800.1
	Mouse1-c*	1024 × 1024 × 56	378.4 × 800.1
	Mouse2	1024 × 1024 × 83	179.7 × 500.2
	Mouse3	1024 × 1024 × 77	250.5 × 500.2
	Mouse4	528 × 391 × 104	209.2 × 839.8
	Mouse5	584 × 498 × 86	345.9 × 839.8
	Mouse6	468 × 443 × 136	283.9 × 839.8
Monkey	Monkey1	1024 × 1024 × 17	240.3 × 1007.0
	Monkey2	1248 × 1248 × 56	177.4 × 420.2
	Monkey3	1248 × 1248 × 68	177.4 × 420.2
	Monkey4	1024 × 1024 × 44	180.2 × 333.3
	Monkey5	1024 × 1024 × 40	198.3 × 999.9
	Monkey6	1024 × 1024 × 45	236.7 × 999.9

^*^ indicates serial histological sections.

**Table 2:** Nuclei segmentation results for the observed mouse and monkey datasets

Dataset	True Positive	False Positive	False Negative	Recall	Precision	F-measure
Mouse1-a*	459	2	17	96.429	99.566	97.972
Mouse1-b*	847	1	16	98.146	99.882	99.006
Mouse1-c*	915	2	29	96.928	99.782	98.334
Mouse2	568	5	20	96.599	99.127	97.847
Mouse3	993	2	19	98.123	99.799	98.954
Mouse4	194	3	20	90.654	98.477	94.404
Mouse5	568	5	22	96.271	98.594	97.594
Mouse6	494	5	19	96.296	98.997	97.268
**Total Mouse**	**5038**	**25**	**162**	**96.180**	**99.322**	**97.726**
Monkey1	695	12	9	98.722	98.303	98.512
Monkey2	754	16	21	97.29	97.922	97.605
Monkey3	605	27	21	96.645	95.728	96.184
Monkey4	561	5	18	96.891	99.117	97.991
Monkey5	580	15	9	98.47	97.479	97.971
Monkey6	604	6	17	97.262	99.016	98.131
**Total Monkey**	**3799**	**81**	**95**	**97.546**	**97.927**	**97.732**

^*^ indicates serial histological sections.

**Table 3:** Actual and predicted classifications result and overall accuracy (number in bold) of cell type computation for mouse and monkey datasets.

		Prediction				Total
		Alpha	Beta	Delta	Unlabeled	
**Mouse**						
Actual	Alpha	490	3	4	2	499
	Beta	0	2146	5	16	2167
	Delta	9	3	135	2	149
	Unlabeled	20	44	11	2021	2096
	Total	519	2196	155	2041	**97.57%**
**Monkey**						
Actual	Alpha	332	5	2	5	344
	Beta	9	1119	4	19	1151
	Delta	2	5	190	3	200
	Unlabeled	24	27	8	2123	2182
	Total	367	1156	204	2150	**97.08%**

**Table 4:** Cell composition of observed mouse and monkey islets.

Dataset	Total nuclei	Number of unlabelled cells	Total alpha cells	Total beta cells	Total delta cells	Total alpha + beta + delta	Percentage alpha (*P*_*α*_)	Percentage beta (*P*_*β*_)	Percentage delta (*P*_*δ*_)
Mouse1-a*	480	278	15	165	22	202	7.4	81.7	10.9
Mouse1-b*	772	343	124	261	44	429	28.9	60.8	10.3
Mouse1-c*	950	621	19	269	41	329	5.8	81.8	12.5
Mouse2	584	59	118	398	9	525	22.5	75.8	1.7
Mouse3	1012	462	146	384	20	550	26.5	69.8	3.6
Mouse4	198	21	32	138	7	177	18.1	78.0	4.0
Mouse5	425	39	23	358	5	386	6.0	92.7	1.3
Mouse6	493	218	46	222	7	275	16.7	80.7	2.5
**Total Mouse**	**4914**	**2041**	**523**	**2195**	**155**	**2873**	**18.2**	**76.4**	**5.4**
Monkey1	700	317	55	309	19	383	14.4	80.7	5.0
Monkey2	770	374	136	170	90	396	34.3	42.9	22.7
Monkey3	630	411	57	126	36	219	26.0	57.5	16.4
Monkey4	587	398	46	128	15	189	24.3	67.7	7.9
Monkey5	579	341	33	172	33	238	13.9	72.3	13.9
Monkey6	610	308	40	251	11	302	13.2	83.1	3.6
**Total Monkey**	**3876**	**2149**	**367**	**1156**	**204**	**1727**	**21.2**	**66.9**	**11.9**
