# Supplementary material for: A novel toolbox to investigate tissue spatial organization applied to the study of the islets of Langerhans
Source: Sci Rep. 2017 Mar 17;7:44261. doi: 10.1038/srep44261 (PMC5355872; doi:10.1038/srep44261)
Supplement: Supplementary Information [file srep44261-s1.pdf]

A novel toolbox to investigate tissue spatial organization applied to the study of the islets of Langerhans

TRAN THI NHU Hoa[1,2+], ARROJO E DRIGO Rafael[3,4+], BERGGREN Per-Olof[3,5*], and BOUDIER Thomas[1,2*]

[1]Sorbonne Universités, UPMC Univ Paris 06, UJF, CNRS, IMT, NUS, Image and Pervasive Access Lab (IPAL), 138632, Singapore
[2]Bioinformatics Institute, Agency for Science, Technology and Research (A*STAR), 138671, Singapore
[3]Lee Kong Chian School of Medicine, Nanyang Technological University, 50 Nanyang Drive, Research Techno Plaza, Level 4, 637 553 Singapore
[4]Nanyang Institute of Structural Biology, Nanyang Technological University, Proteos, 61 Biopolis Drive, 138673, Singapore
[5]Rolf Luft Research Center for Diabetes and Endocrinology, Karolinska Institutet, Stockholm, Sweden
[*]Corresponding authors: thomas.boudier@upmc.fr, per-olof.berggren@ki.se
[+]Equal contribution

ABSTRACT

Supplementary Information

Dataset	Total number of direct interactions	Maximum number of direct interactions for one cell	Average and standard deviation of number of direct interactions, per cell	Avg. and sd of number of direct interactions for alpha cells	Avg. and sd of number of direct interactions for beta cells	Avg. and sd of number of direct interactions for delta cells
Mouse1-a	370	7	3.66 ± 1.44	3.13 ± 1.09	3.76 ± 1.40	3.32 ± 1.74
Mouse1-b	1206	11	5.622 ± 1.82	4.79 ± 2.06	5.96 ± 1.55	5.98 ± 1.84
Mouse1-c	747	9	4.54 ± 1.47	3.79 ± 1.64	4.59 ± 1.35	4.56 ± 1.98
Mouse2	2064	15	7.86 ± 2.35	7.63 ± 2.51	7.9 ± 2.3	7.78 ± 2.09
Mouse3	1856	16	6.75 ± 2.39	7.19 ± 3.03	6.42 ± 1.88	9.75 ± 3.05
Mouse4	662	15	7.48 ± 3.03	6.63 ± 2.71	7.70 ± 3.08	7.00 ± 2.62
Mouse5	1734	20	8.98 ± 3.03	6.69 ± 2.83	9.13 ± 2.99	8.60 ± 1.74
Mouse6	1368	18	9.94 ± 3.26	7.43 ± 2.55	10.45 ± 3.15	10.28 ± 3.28
Monkey1	850	9	4.44 ± 1.61	3.78 ± 1.60	4.63 ± 1.56	3.32 ± 1.38
Monkey2	804	11	4.06 ± 1.76	4.06 ± 1.60	3.81 ± 1.54	4.53 ± 2.23
Monkey3	412	11	3.76 ± 2.10	4.35 ± 2.26	3.39 ± 1.98	4.11 ± 1.99
Monkey4	417	9	4.41 ± 1.69	4.19 ± 1.73	4.39 ± 1.64	5.2 ± 1.87
Monkey5	430	8	3.61 ± 1.46	3.51 ± 1.67	3.47 ± 1.40	4.45 ± 1.25
Monkey6	677	9	4.48 ± 1.62	4.57 ± 1.43	4.43 ± 1.66	5.27 ± 1.05

Supplementary Table 1. Number of direct interactions, per cell, for all datasets.

Type interaction	alpha-alpha	beta-beta	delta-delta	alpha-beta	alpha-delta	beta-delta	Total
Mouse1-a	7	282	16	24	9	32	370
Mouse1-b	142	623	50	228	82	81	1206
Mouse1-c	7	566	45	32	26	71	747
Mouse2	228	1354	2	414	30	36	2064
Mouse3	332	1070	17	276	110	51	1856
Mouse4	53	468	6	98	8	29	662
Mouse5	19	1558	2	116	0	39	1734
Mouse6	61	1037	2	200	20	48	1368
Total mouse	849	6958	140	1388	285	387	10007
**Average (%)**	**8.48**	**69.53**	**1.4**	**13.87**	**2.85**	**3.87**	**100**
Monkey1	37	631	6	125	9	42	850
Monkey2	162	218	143	159	69	53	804
Monkey3	55	152	29	86	52	38	412
Monkey4	45	227	9	76	27	33	417
Monkey5	13	235	36	71	19	56	430
Monkey6	24	476	2	121	14	40	677
Total monkey	336	1939	225	638	190	262	3590
**Average (%)**	**9.35**	**54.01**	**6.27**	**17.77**	**5.29**	**7.31**	**100**

Supplementary Table 2. Number of direct interactions between different islet-cell types in observed mouse and monkey islets.

Cell Distance	d=1	d=2	d=3	d=4	d=5	d=6	d=7	d=8	d>=9
Observed Mouse1-a	20	28	29	31	13	13	11	11	9
Simulated Mouse1-a	40.52	42.15	32.79	20.93	11.67	6.79	4.18	2.7	3.27
Observed Mouse1-b	139	91	30	1	0	0	0	0	0
Simulated Mouse1-b	214.93	41.54	3.82	0.52	0.15	0.04	0	0	0
Observed Mouse1-c	25	32	42	43	31	36	31	16	13
Simulated Mouse1-c	60.91	72.62	60.31	39.03	20.32	8.89	3.97	1.76	1.19
Observed Mouse2	169	120	81	28	0	0	0	0	0
Simulated Mouse2	336.81	60.26	0.93	0	0	0	0	0	0
Observed Mouse3	125	86	61	43	32	21	12	4	0
Simulated Mouse3	325.57	55.83	2.5	0.1	0	0	0	0	0
Observed mouse4	50	58	20	2	0	0	0	0	0
Simulated mouse4	101.11	26.88	1.86	0.15	0	0	0	0	0
Observed Mouse5	74	85	77	69	46	5	1	0	0
Simulated Mouse5	149.56	147.25	47.99	10.36	1.64	0.2	0	0	0
Observed Mouse6	93	94	34	1	0	0	0	0	0
Simulated Mouse6	181.43	39.5	1.06	0.01	0	0	0	0	0
Observed monkey1	94	94	63	39	16	1	0	0	0
Simulated monkey1	148.23	100.48	40.21	12.43	3.69	1.07	0.49	0.23	0.17
Observed Monkey2	85	38	19	10	6	4	1	0	0
Simulated monkey2	128.6	28.82	4.62	0.81	0.12	0.02	0.01	0	0
Observed Monkey3	25	15	10	4	1	0	0	0	0
Simulated monkey3	37.93	13.73	2.62	0.57	0.13	0.02	0	0	0
Observed Monkey4	48	32	24	17	6	1	0	0	0
Simulated monkey4	87.42	33.19	6.53	0.79	0.05	0.02	0	0	0
Observed Monkey5	52	40	27	21	16	5	3	2	3
Simulated Monkey5	68.39	51.49	27.24	12.39	5.4	2.04	0.96	0.55	0.77
Observed Monkey6	89	74	45	20	13	6	1	0	0
Simulated Monkey6	114.19	81.53	35	11.94	3.56	1.1	0.38	0.16	0.1

Supplementary Table 3. Histogram of cell distances between alpha and beta cells in observed and simulated - average of 100 simulated random organization for mouse and monkey islets, d : distance unit.

Dataset	SDI F Euclidean Distance	SDI G Euclidean Distance	SDI F Cell Distance	SDI G Cell Distance	SDI F Cluster	SDI G Cluster
Mouse1-a	0.99	0	0.99	0	0.31	1
Mouse1-b	1	0	1	0.805	0.08	1
Mouse1-c	0.99	0.63	1	0.02	0.97	0.98
Mouse2	1	0	1	0	0.72	0.96
Mouse3	1	0	1	0	0.97	0.82
Mouse4	1	0	1	0	1	0
Mouse5	1	0	1	0	0.96	0.81
Mouse6	1	0	1	0.01	0.62	0.98
Mouse4 randomized	0.91	0.62	0.83	0.38		
Monkey1	1	0	1	0	0.85	0.8
Monkey2	1	0	1	0	0.02	0.96
Monkey3	1	0	1	0	0.84	1
Monkey4	0.99	0.01	1	0.01	0.22	0.705
Monkey5	0.96	0.01	0.98	0.005	0.74	0.96
Monkey6	1	0.01	1	0	0.19	0.96
Monkey1 randomized	0.1	0.64	0.44	0.72		

Supplementary Table 4. Spatial descriptor index (SDI) for F-function and G-function, calculated using Euclidean distance and cell distance.

Dataset	Number of clusters	Average number of cells in cluster	Maximum number of cells in cluster
Mouse1-a	8	1.88	3
Mouse1-b	39	3.18	27
Mouse1-c	13	1.46	3
Mouse2	16	7.38	34
Mouse3	20	7.3	93
Mouse4	7	4.57	10
Mouse5	7	3.3	10
Mouse6	6	7.67	36
**Total Mouse**	**116**	**5.43**	
Monkey1	25	2.2	16
Monkey2	34	4	33
Monkey3	15	3.8	22
Monkey4	13	3.53	27
Monkey5	20	1.65	4
Monkey6	21	1.9	8
**Total Monkey**	**128**	**2.85**	

Supplementary Table 5. Cluster analysis for all datasets, with number of clusters in each dataset, average number of cells in clusters and maximum number of cells in a cluster.

Model / Frequency	$P_{\alpha\alpha}$	$P_{\beta\beta}$	$P_{\delta\delta}$	$P_{\alpha\beta}$	$P_{\alpha\delta}$	$P_{\beta\delta}$
Observed Mouse1-a	0.026	0.712	0.041	0.081	0.033	0.107
Theoretical Mouse1-a	0.01	0.615	0.014	0.155	0.023	0.183
Simulated Mouse1-a	0.011	0.61	0.016	0.154	0.022	0.187
Observed Mouse1-b	0.121	0.505	0.03	0.206	0.067	0.07
Theoretical Mouse1-b	0.096	0.342	0.011	0.363	0.064	0.122
Simulated Mouse1-b	0.093	0.34	0.012	0.356	0.068	0.131
Observed Mouse1-c	0.002	0.661	0.126	0.024	0.028	0.158
Theoretical Mouse1-c	0.001	0.537	0.052	0.056	0.017	0.336
Simulated Mouse1-c	0.001	0.525	0.067	0.059	0.019	0.329
Observed Mouse2	0.098	0.651	3.77E-004	0.222	0.014	0.016
Theoretical Mouse2	0.059	0.548	0	0.358	0.009	0.028
Simulated Mouse2	0.058	0.547	3.46E-004	0.359	0.009	0.027
Observed Mouse3	0.205	0.53	0.005	0.192	0.047	0.021
Theoretical Mouse3	0.105	0.417	0.001	0.419	0.019	0.039
Simulated Mouse3	0.103	0.422	7.28E-004	0.416	0.019	0.039
Observed Mouse4	0.046	0.712	0.009	0.155	0.026	0.052
Theoretical Mouse4	0.029	0.612	0.002	0.266	0.016	0.075
Simulated Mouse4	0.0287	0.607	0.0024	0.269	0.017	0.0759
Observed Mouse5	0.011	0.899	0.001	0.067	0	0.022
Theoretical Mouse5	0.004	0.86	1.678E-4	0.111	0.002	0.024
Simulated Mouse5	0.004	0.86	1.384E-4	0.112	0.001	0.023
Observed Mouse6	0.045	0.758	0.001	0.146	0.015	0.035
Theoretical Mouse6	0.028	0.652	6.479E-4	0.27	0.009	0.041
Simulated Mouse6	0.027	0.653	6.579E-4	0.272	0.009	0.04
Observed Monkey1	0.03	0.736	0.008	0.141	0.011	0.074
Theoretical Monkey1	0.019	0.653	0.003	0.223	0.015	0.087
Simulated Monkey1	0.0186	0.6455	0.0024	0.2255	0.016	0.092
Observed Monkey2	0.17	0.243	0.169	0.207	0.111	0.1
Theoretical Monkey2	0.11	0.172	0.064	0.276	0.167	0.209
Simulated Monkey2	0.112	0.173	0.063	0.278	0.165	0.21
Observed Monkey3	0.159	0.341	0.075	0.216	0.111	0.099
Theoretical Monkey3	0.071	0.329	0.025	0.307	0.085	0.183
Simulated Monkey3	0.117	0.347	0.041	0.223	0.094	0.179
Observed Monkey4	0.094	0.518	0.026	0.192	0.081	0.088
Theoretical Monkey4	0.053	0.453	0.009	0.311	0.044	0.128
Simulated Monkey4	0.05	0.461	0.008	0.31	0.042	0.129
Observed Monkey5	0.03	0.547	0.084	0.165	0.044	0.13
Theoretical Monkey5	0.019	0.522	0.019	0.2	0.038	0.2
Simulated Monkey5	0.017	0.517	0.025	0.199	0.04	0.203
Observed Monkey6	0.035	0.703	0.003	0.179	0.021	0.059
Theoretical Monkey6	0.018	0.691	0.001	0.22	0.01	0.061
Simulated Monkey6	0.018	0.688	0.002	0.22	0.01	0.062

Supplementary Table 6. Observed - raw data and theoretical frequencies - theoretical random model [Hoang et al.] and average of 100 simulated random organizations for direct interaction (upon random model) between different islet-cell types in mouse and monkey islets.

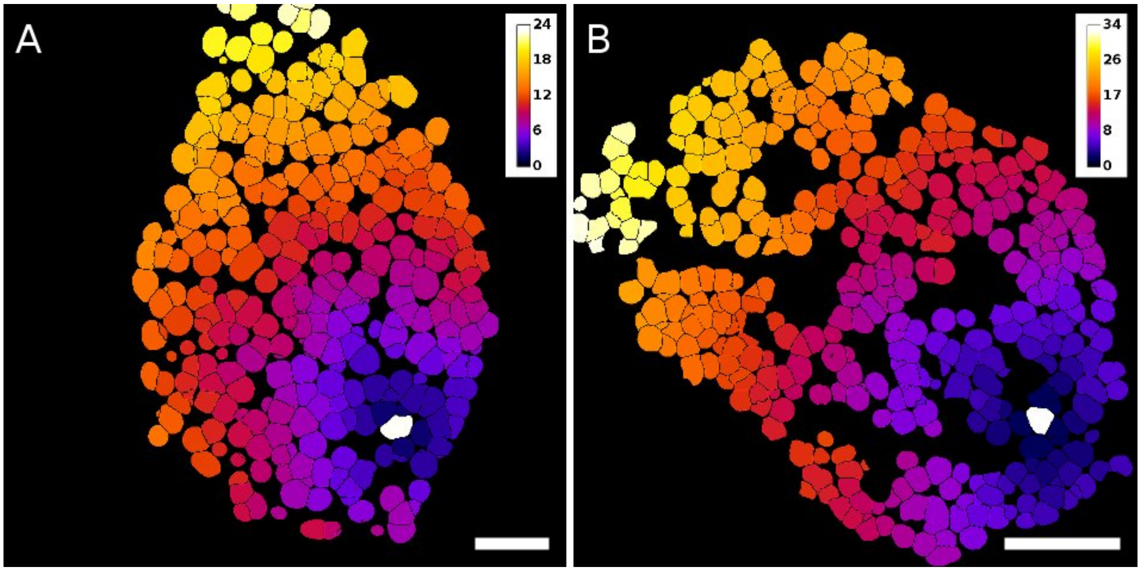

Supplementary Figure 1. Result of cell distance computation in Mouse1-b and Monkey1 islet, one slice is displayed, the distance is expressed as cell distance between all cells and a reference cell (displayed in white). Reference cell has distance 0, a given islet-cell "X" contacts directly a reference cell, "X" (dark purple cells) will be at distance equal to 1 from the reference cell. If another islet-cell "Y" contacts islet cell "X", "Y" will then be at a distance value of 2 from the "reference" cell, and so on. Scale bar, 50 μm.

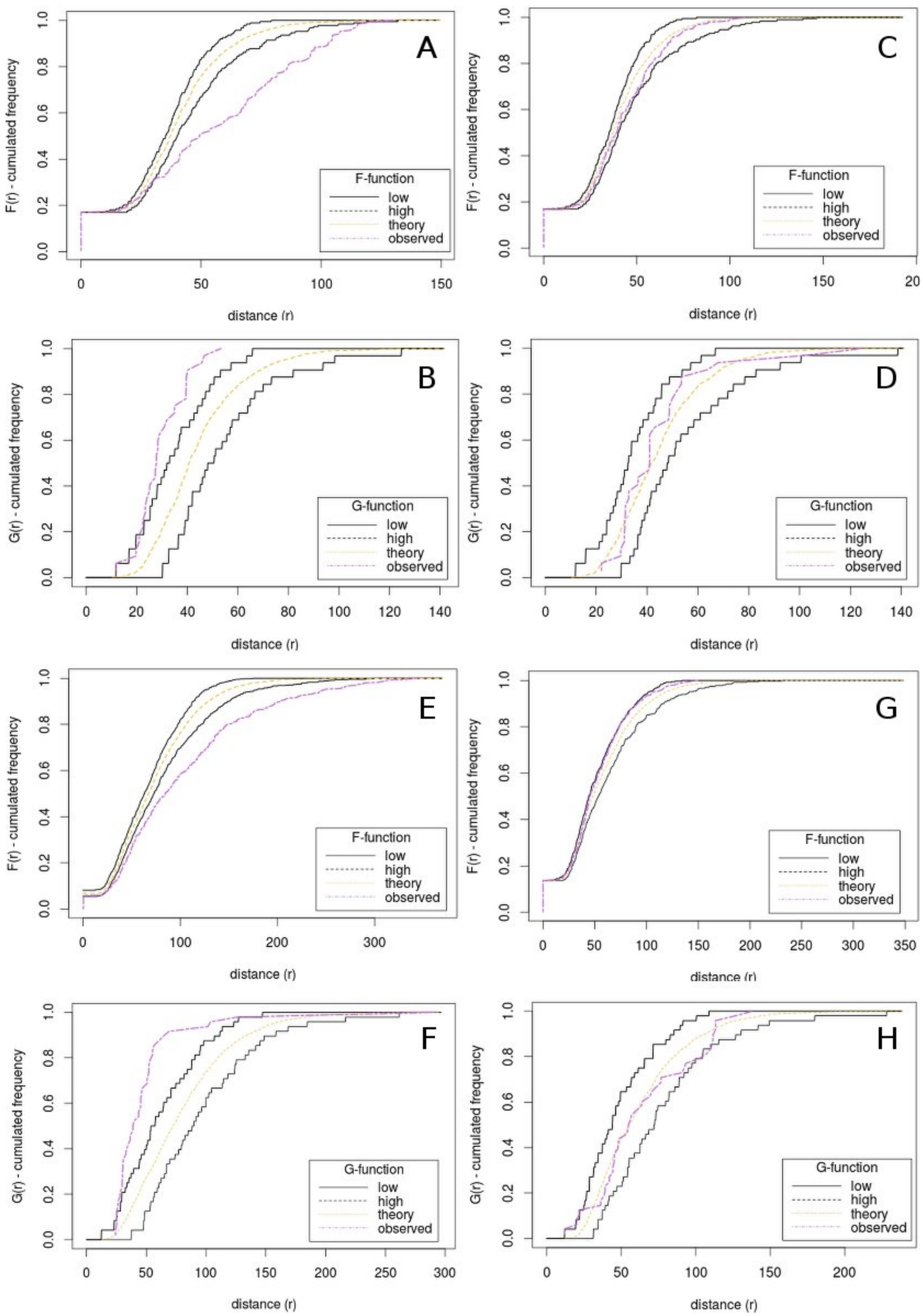

Supplementary Figure 2. The corresponding F-function, G-function cumulative distribution functions of the observed data and simulated random organization for alpha-cells within mouse4 and monkey1 islets, using Euclidean distance. F-function (A) and G-function (B) of observed mouse4 islet, F-function (C) and G-function (D) of simulated random organization of mouse4 islet. F-function (E) and G-function (F) of observed monkey1 islet, F-function (G) and G-function (H) of simulated random organization of monkey1 islet. In black (low) low envelope of 5% confidence interval, in black (high) high envelope of 95% confidence interval, in orange (theory) average value of 100 simulated random organization, in violet (observed), our observed data. Note that for observed data the curve is outside interval delimited by the 5%-95% confidence interval.

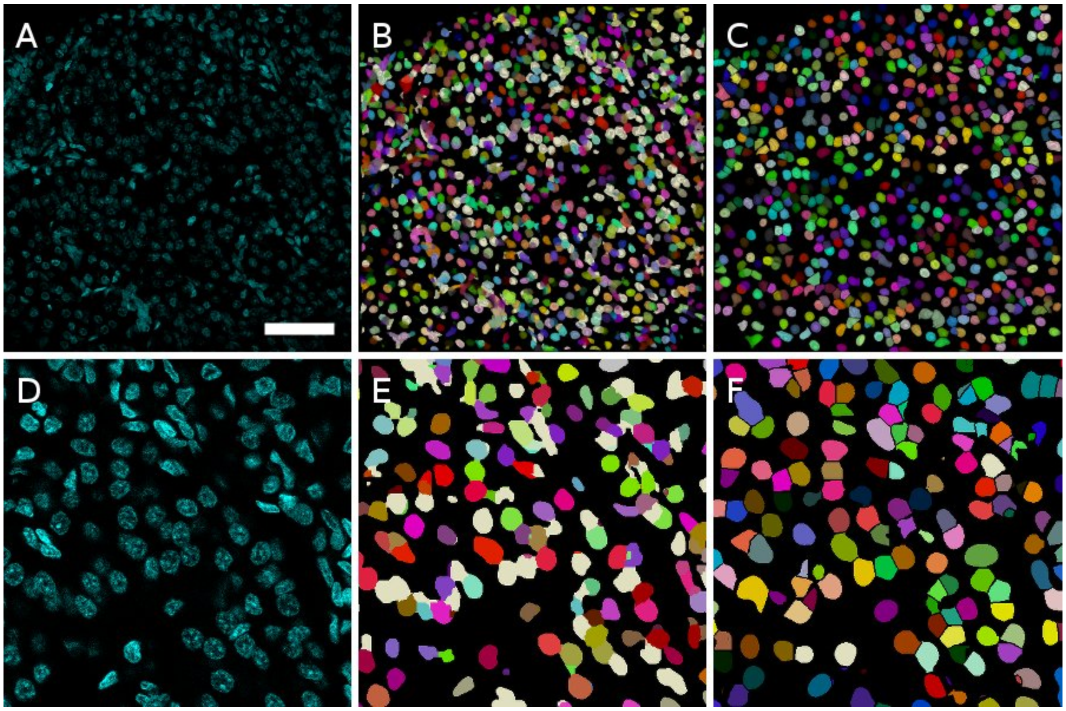

Supplementary Figure 3. Comparison of our nuclei segmentation result with related segmentation tool - FARSIGHT. Top: 3D raw data (A) and 3D output generated by FARSIGHT (B), by our algorithm (C), volume rendering using ImageJ 3DViewer. Bottom: visualization of zoom in 2D section of the same data, raw data (D), output generated by FARSIGHT (E), by our algorithm (F). Scale bar, 100 μm. For monkey islet, FARSIGHT gives 1539 segmented nuclei / 695 true nuclei. However, our algorithm gives 714 segmented nuclei. The accuracy of our algorithm is about 97%, however the accuracy of FARSIGHT is lower and below 80%.
